# High triglyceride–glucose index is associated with poor prognosis in patients with acute ST-elevation myocardial infarction after percutaneous coronary intervention

**DOI:** 10.1186/s12933-019-0957-3

**Published:** 2019-11-13

**Authors:** Erfei Luo, Dong Wang, Gaoliang Yan, Yong Qiao, Bo Liu, Jiantong Hou, Chengchun Tang

**Affiliations:** 1 School of Medicine, Southeast University, Nanjing, 210009 China; 2 Department of Cardiology, Zhongda Hospital, Southeast University, Hunan Road, Nanjing, 210009 Jiangsu China

## Abstract

**Background:**

Insulin resistance (IR) is considered a pivotal risk factor for cardiometabolic diseases, and the triglyceride–glucose index (TyG index) has emerged as a reliable surrogate marker of IR. Although several recent studies have shown the association of the TyG index with vascular disease, no studies have further investigated the role of the TyG index in acute ST-elevation myocardial infarction (STEMI). The objective of the present study was to evaluate the potential role of the TyG index as a predictor of prognosis in STEMI patients after percutaneous coronary intervention (PCI).

**Methods:**

The study included 1092 STEMI patients who underwent PCI. The patients were divided into 4 quartiles according to TyG index levels. Clinical characteristics, fasting plasma glucose (FPG), triglycerides (TGs), other biochemical parameters, and the incidence of major adverse cardiovascular and cerebral events (MACCEs) during the follow-up period were recorded. The TyG index was calculated using the following formula: ln[fasting TGs (mg/dL) × FPG (mg/dL)/2].

**Results:**

The incidence of MACCEs and all-cause mortality within 30 days, 6 months and 1 year after PCI were higher among STEMI patients with TyG index levels in the highest quartile. The TyG index was significantly associated with an increased risk of MACCEs in STEMI patients within 1 year after PCI, independent of confounding factors, with a value of 1.529 (95% CI 1.001–2.061; P = 0.003) for those in the highest quartile. The area under the curve (AUC) of the TyG index predicting the occurrence of MACCEs in STEMI patients after PCI was 0.685 (95% CI 0.610–0.761; P = 0.001). The results also revealed that Killip class > 1, anaemia, albumin, uric acid, number of stents and left ventricular ejection fraction (LVEF) were independent predictors of MACCEs in STEMI patients after PCI (all P < 0.05).

**Conclusions:**

This study indicated an association between higher TyG index levels and increased risk of MACCEs in STEMI patients for the first time, and the TyG index might be a valid predictor of clinical outcomes in STEMI patients undergoing PCI.

*Trial Registration* ChiCTR1900024577.

## Background

Acute coronary syndrome (ACS) remains a leading cause of morbidity and mortality. In the USA alone, more than 1,000,000 suffer from ACS annually [1]. The Global Registry of Acute Coronary Events (GRACE) study showed that the mortality rate of ACS patients after 1 year is approximately 15%, and the cumulative mortality rate after 5 years is as high as 20% [2]. Consequently, early risk stratification is important to prevent and manage ACS [3, 4]. Insulin resistance (IR), the decreased insulin sensitivity of peripheral tissues characterized by defects in the uptake and oxidation of glucose, plays a critical role in the pathogenesis of diabetes as well as cardiovascular disease (CVD) [5]. The molecular mechanisms include the roles of IR in vascular function, macrophage accumulation, atherosclerosis development and hypertension [6, 7]. Previous studies have noted that the hyperinsulinaemic-euglycaemic clamp is the gold standard diagnostic method for IR. However, the technique is difficult to implement in large epidemiological investigations because it is costly, time consuming and complex [8]. Recently, the triglyceride–glucose index (TyG index), the product of fasting plasma glucose (FPG) and triglycerides (TGs), has been used in clinical practice as a simple and reliable surrogate marker of IR [9, 10]. In addition, the TyG index has been shown to be well correlated with the homeostasis model assessment of insulin resistance (HOMA-IR) and hyperinsulinaemic-euglycaemic clamp [11–13]. Zhang et al. showed that the cumulative risk of incident type 2 diabetes mellitus (DM) increased with the TyG index [14]. In addition, previous studies have shown that the TyG index is associated with coronary artery calcification, carotid atherosclerosis, symptomatic coronary artery disease and a high risk of CVD [15–18]. Moreover, Sanchez et al. showed associations of the highest TyG index values with the incidence of stroke and hypertension and that the TyG index may predict the development of cardiovascular events [19–21]. To the best of our knowledge, the relationship between TyG index levels and clinical outcomes in patients with ST-segment elevation myocardial infarction (STEMI) undergoing percutaneous coronary intervention (PCI) has not been fully evaluated. The purpose of this study was to explore the predictive value of TyG index levels on the clinical outcomes of STEMI patients after PCI and to provide ideas for improving STEMI risk stratification.

## Methods

### Study population

This study is a retrospective observational cohort study. From January 2012 to March 2018, consecutive patients with STEMI admitted to Zhongda Hospital (Nanjing, People’s Republic of China) were enrolled. The inclusion criteria were as follows: (1) 18 ≤ age ≤ 80 years old and (2) diagnosis of STEMI based on the Guidelines for the Diagnosis and Treatment of Acute ST-segment Elevation Myocardial Infarction in 2010 (China) [22] and treatment with PCI. The exclusion criteria were as follows: a history of major surgery, trauma or bleeding over the past 3 months; malignant tumour; serious injury of liver or kidney; contraindications to anticoagulant and antiplatelet therapy; and incomplete clinical data and coronary angiography.

### Grouping

The patients were divided into 4 quartiles according to TyG index levels, Q1 (n = 273, TyG index ≤ 8.691), Q2 (n = 273, 8.692 ≤ TyG index ≤ 9.097), Q3 (n = 273, 9.098 ≤ TyG index ≤ 9.607), and Q4 (n = 273, TyG index ≥ 9.608).

### Laboratory investigations

Blood samples were obtained from each patient from the cubital vein after an overnight fast ≥ 12 h. Concentrations of FPG were measured by the enzymatic hexokinase method. TGs, total cholesterol (TC), high-density lipoprotein–cholesterol (HDL-C), and low-density lipoprotein–cholesterol (LDL-C) were measured using an automatic biochemistry analyser (Hitachi 7150, Japan) in an enzymatic assay. The TyG index was calculated using the following formula: ln [fasting TGs (mg/dL) × FPG (mg/dL)/2] [23].

### Percutaneous coronary intervention

PCI includes balloon dilation and/or stent implantation for infarct-related vessels and was performed by experienced operators according to standard techniques. All patients were given aspirin (300 mg), ticagrelor (180 mg) or clopidogrel (300 mg) before surgery, and aspirin (100 mg, QD), ticagrelor (90 mg, BID) or clopidogrel (75 mg, QD) were administered after surgery. Statins, nitrates, beta blockers, and angiotensin-converting enzyme inhibitors were commonly used in all patients without contraindications.

### Endpoints and definitions

The endpoints were major adverse cardiac and cerebrovascular events (MACCEs) during the follow-up period (30 days, 6 months and 1 year after PCI). The MACCEs included all-cause death, target vessel revascularization, myocardial infarction during follow-up, unstable angina pectoris requiring hospitalization, heart failure, stroke or transient cerebral ischaemia.

Hypertension was defined as a self-reported physician-diagnosed condition, currently use of antihypertensive treatment, and/or systolic blood pressure (SBP) ≥ 140 mm Hg and/or diastolic blood pressure (DBP) ≥ 90 mmHg [24]. Diabetes was defined according to one of the following criteria: (1) self-reported diabetes that was previously diagnosed by a physician or the use of glucose-lowering drugs before hospitalization; (2) any one of the characteristic symptoms of DM such as thirst, polyuria, polyphagia, and weight loss with any blood glucose estimation exceeding 11.1 mmol/L; (3) a fasting blood glucose level in excess of 7.0 mmol/L after an overnight fast of 8 h; and (4) a 2-h blood glucose estimation exceeding 11.1 mmol/L after a 75 g glucose load via an oral glucose tolerance test after an overnight fast of 8 h [25].

Each patient’s baseline clinical data, including sex, age, height, weight, heart rate, SBP, DBP, and Killip class, as well as previous and personal histories, including hypertension, diabetes, atrial fibrillation, anaemia, previous myocardial infarction (MI) and smoking history, were recorded. Haematological examination indexes, including white blood cells, neutrophil-to-platelet ratio, albumin, measured HbA1c, high-sensitivity C-reactive protein (hs-CRP), uric acid, estimated glomerular filtration rate (eGFR), cardiac troponin I, N-terminal proB-type natriuretic peptide (NT-proBNP), echocardiography parameters, medications, and coronary angiography data were recorded. Killip classification was a useful tool for risk stratification. Killip class I was defined by the absence of signs of pulmonary congestion or systemic hypoperfusion. Killip class II was defined by the presence of rales in the lower half of the lung fields or by the presence of gallop heart sounds; Killip class III was defined by the presence of rales in the upper half of the lung fields; and Killip class IV was characterized by cardiogenic shock (significant hypotension: SBP < 90 mm Hg or requiring inotropes) [26]. Body mass index (BMI) was calculated as the body mass divided by the square of the body height and expressed in units of kg/m^2^. Blood pressure (BP) was measured by experienced physicians using an automated BP monitor (HEM-7080IC; Omron Healthcare, Lake Forest, IL, USA). Patients were seated for at least 10 min in a quiet environment with their feet on the floor and their arm supported at heart level. The average of 3 consecutive BP measurements taken at 2-min intervals on the same arm was recorded for the study. The Gensini score of each patient was calculated according to the results of coronary angiography. Follow-up data were obtained from hospital records or via interviews (in person or by telephone) of patients and their families conducted by at least two cardiologists.

### Statistical analysis

Analyses were performed using SPSS software, version 19.0 (SPSS, Inc., Chicago, IL, USA). Continuous variables are expressed as the mean ± SD or median (inter-quartile range). Categorical variables are reported in frequencies with percentages. Univariate and multivariate logistic regression analyses were used to identify MACCE predictors. Variables with univariate P values < 0.10 were selected for multivariate analysis and are expressed as odds ratios (ORs) with 95% confidence intervals (CIs). Survival was graphically represented using Kaplan–Meier curves. Differences in survival rates were compared using the log-rank test. The area under the receiver operating characteristic (ROC) curves (AUCs) was used to indicate the predictive value of the TyG index for MACCEs. All tests were 2-tailed, and statistical significance was defined as a P-value < 0.05.

## Results

### Patient characteristics

Briefly, 1178 individuals fulfilled the inclusion criteria. A group of 32 patients had missing laboratory values, and 54 did not complete the follow-up. These restrictions left 1092 participants available for the final baseline analysis. The study population had an average age of 62.4 ± 12.5 years and an average BMI of 25.2 ± 2.2 kg/m^2^; 864 (79.1%) were male. A total of 678 patients (62.1%) had a history of hypertension, and 270 patients (24.7%) had a history of DM. All patients were subdivided into 4 groups according to TyG index levels. The baseline characteristics of the 4 groups are shown in Table 1. There were statistically significant differences (P < 0.05) among the four groups in terms of age, BMI, SBP, DBP, heart rate, hypertension, DM, platelets, albumin, FPG, measured HbA1c, TGs, TC, HDL-C, LDL-C, uric acid, three-vessel disease, and Gensini score, and no statistically significant differences were found in the other indicators. The TyG index was positively correlated with BMI, SBP, DBP, heart rate, platelets, albumin, FPG, measured HbA1c, TGs, TC, HDL-C, LDL-C, uric acid, and Gensini score, while it was negatively related to age. Patients with a high TyG index had a higher incidence of hypertension (P < 0.001), DM (P < 0.001) and three-vessel disease (P < 0.001).

**Table 1:** Baseline characteristics of 4 groups

Variable	Q1 (n = 273)	Q2 (n = 273)	Q3 (n = 273)	Q4 (n = 273)	P value
TyG index	8.373 ± 0.258	8.905 ± 0.122	9.345 ± 0.145	10.076 ± 0.483	< 0.001
Age, years	65.2 ± 13.2	63.5 ± 10.8	61.2 ± 11.6	57.6 ± 12.4	< 0.001
Male	219 (80.2)	220 (80.6)	222 (81.3)	213 (78.0)	0.546
BMI, kg/m2	25.2 (24.7–25.5)	25.5 (25.1–25.8)	26.0 (25.6–26.2)	26.5 (26.0–26.9)	0.012
SBP, mmHg	124.9 ± 22.1	128.7 ± 21.9	129.9 ± 20.8	138.3 ± 22.6	< 0.001
DBP, mmHg	75.2 ± 14.1	76.6 ± 13.4	77.1 ± 14.3	84.7 ± 16.8	< 0.001
Heart rate, bpm	77.7 ± 14.5	79.7 ± 13.1	79.0 ± 12.7	82.2 ± 16.3	0.019
Killip class > 1	82 (30.3)	85 (31.1)	91 (33.3)	89 (32.6)	0.523
Smoker	114 (41.8)	120 (44.0)	123 (45.1)	126 (46.2)	0.886
Hypertension	138 (50.5)	162 (60.0)	177 (64.8)	201 (73.6)	< 0.001
Diabetes mellitus	21 (7.7)	45 (16.5)	81 (29.7)	123 (45.1)	< 0.001
Anemia	33 (12.1)	26 (9.5)	30 (11.0)	35 (12.8)	0.322
Previous AMI	6 (2.2)	6 (2.2)	7 (2.6)	8 (2.9)	0.489
Atrial fibrillation	16 (5.8)	13 (4.8)	17 (6.2)	15 (5.5)	0.652
Biochemical indicators					
NT-proBNP, pg/mL (IQR)	525.5 (31.0–2193.2)	534.4 (53.5–2293.7)	527.6 (34.9–2401.4)	546 (71.2–2603.2)	0.179
Cardiac troponin I, ng/ml (IQR)	12.2 (3.01–22.7)	11.6 (2.7–23.1)	13.1 (2.3–23.9)	12.7 (2.6–24.2)	0.762
hs-CRP, mg/L (IQR)	7.9 (5.5–10.4)	7.3 (4.4–11.1)	6.9 (3.9–9.7)	7.3 (3.4–10.7)	0.652
White blood cells, 10^9^/L	9.9 ± 3.9	10.0 ± 3.9	10.0 ± 3.2	10.2 ± 3.2	0.826
Neutrophil ratio	75.5 ± 10.3	76.2 ± 11.4	75.2 ± 13.6	75.9 ± 12.9	0.775
Platelet, 10^9^/L	195.0 ± 56.1	201.8 ± 60.5	228.8 ± 63.0	223.8 ± 53.9	< 0.001
Albumin, g/L	36.1 ± 4.8	37.3 ± 4.1	37.6 ± 4.5	38.2 ± 5.2	0.022
FPG, mmol/L	6.6 ± 1.9	7.1 ± 1.8	8.1 ± 2.4	11.7 ± 6.0	< 0.001
Measured HbA1c, % (IQR)	6.5 (6.2–6.8)	7.1 (6.9–7.4)	7.6 (7.5–7.8)	8.0 (7.9–8.2)	< 0.001
TC, mmol/L	4.0 ± 0.9	4.3 ± 1.0	4.8 ± 1.2	5.2 ± 1.3	< 0.001
Triglycerides, mmol/L	0.9 ± 0.3	1.4 ± 0.3	1.9 ± 0.6	3.6 ± 2.6	< 0.001
HDL-C, mmol/L	1.0 ± 0.3	1.1 ± 0.2	1.2 ± 0.3	1.2 ± 0.3	< 0.001
LDL-C, mmol/L	2.4 ± 0.7	2.7 ± 0.8	3.0 ± 1.0	3.1 ± 0.8	< 0.001
Uric acid, umol/L	319.5 ± 102.6	325.5 ± 87.3	342.2 ± 109.3	347.4 ± 100.6	0.019
eGFR, mL/min	87.0 ± 32.8	85.4 ± 42.0	86.9 ± 34.3	89.5 ± 39.1	0.423
Coronary angiography					
GPIIb/IIIa antagonists	91 (33.3)	89 (32.6)	86 (31.8)	92 (33.7)	0.644
Anticoagulation	25 (9.2)	27 (9.9)	22 (8.1)	24 (8.8)	0.546
Lesion vessels	3.2 ± 1.6	3.2 ± 1.5	3.0 ± 1.8	3.1 ± 1.3	0.743
Three-vessel disease	105 (38.5)	138 (50.5)	150 (54.9)	168 (61.5)	< 0.001
Number of stents	1.03 ± 0.54	1.02 ± 0.47	1.02 ± 0.45	1.11 ± 0.50	0.114
Gensini score, (IQR)	61.6 (49.3–75.7)	65.4 (54.1–72.6)	71.6 (60.9–79.3)	78.3 (63.1–83.9)	0.049
Echocardiography					
LVEF	0.55 ± 0.12	0.54 ± 0.10	0.56 ± 0.10	0.55 ± 0.12	0.335
Medications use at discharge					
Aspirin	255 (93.4)	264 (96.7)	264 (96.7)	267 (97.8)	0.059
Clopidogrel/Ticagrelor	267 (97.8)	270 (98.9)	267 (97.8)	273 (100)	0.082
Statin	261 (95.6)	265 (97.1)	267 (97.8)	260 (95.2)	0.321
Beta blockers	215 (78.8)	228 (83.5)	210 (76.4)	223 (81.1)	0.247
ACEI/ARB	168 (61.1)	180 (65.5)	171 (62.2)	183 (66.5)	0.205

Data are presented as the IQR, mean ± SD or n (%)

*BMI* body mass index, *SBP* systolic blood pressure, *DBP* diastolic blood pressure, *AMI* acute myocardial infarction, *IQR* interquartile range, *hs-CRP* hypersensitive C-reactive protein, *FPG* fasting plasma glucose, *TC* total cholesterol, *HDL-C* high-density lipoprotein cholesterol, *LDL-C* low-density lipoprotein cholesterol, *SCr* Serum creatinine concentration, *eGFR* estimated glomerular filtration rate, *LVEF* left ventricular ejection fraction

### Risk factors for MACCEs

The baseline characteristics of the MACCE and MACCE-free groups are shown in Table 2. The prevalence of Killip class > 1, smoking history, hypertension, DM, anaemia, lesion vessels, number of stents and three-vessel disease of the MACCE group were higher than that of the MACCE-free group (all P < 0.05). There were statistically significant differences (P < 0.05) between the MACCE and MACCE-free groups in terms of age, DBP, white blood cells, platelets, albumin, FPG, measured HbA1c, uric acid, eGFR and left ventricular ejection fraction (LVEF).

**Table 2:** Baseline characteristics of the MACCE and MACCE-free groups

Variable	MACCE group (n = 375)	MACCE-free group (n = 717)	P value
Age, years	64.5 ± 12.5	61.3 ± 12.4	< 0.001
Male	300 (80.0)	564 (78.7)	0.638
BMI, kg/m^2^	25.3 ± 2.1	25.1 ± 2.4	0.382
SBP, mmHg	128.7 ± 18.9	128.8 ± 22.7	0.939
DBP, mmHg	76.1 ± 13.9	78.0 ± 14.1	0.028
Heart rate, bpm	79.5 ± 15.4	79.7 ± 14.5	0.905
Killip class > 1	142 (37.9)	210 (29.3)	< 0.001
Smoker	199 (53.1)	317 (44.2)	0.012
Hypertension	261 (69.6)	417 (58.2)	< 0.001
Diabetes mellitus	117 (31.2)	153 (21.3)	< 0.001
Anemia	67 (17.9)	74 (10.3)	0.023
Previous AMI	9 (2.4)	15 (2.1)	0.447
Atrial fibrillation	15 (4.0)	33 (4.6)	0.385
Biochemical indicators			
NT-proBNP, pg/mL (IQR)	525.5 (31.0–2193.2)	546.8 (71.2–2603.2)	0.179
Cardiac troponin I, ng/ml	13.1 (3.18–23.5)	12.7 (2.8–23.2)	0.480
hs-CRP, mg/L (IQR)	7.6 (4.6–9.8)	7.1 (3.2–10.1)	0.152
White blood cells, 10^9^/L	10.4 ± 3.6	9.9 ± 3.4	0.046
Neutrophil ratio	75.6 ± 12.8	75.8 ± 11.8	0.680
Platelet, 10^9^/L	217.2 ± 68.6	208.4 ± 56.8	0.024
Albumin, g/L	36.4 ± 5.7	37.1 ± 4.5	0.021
FPG, mmol/L	9.0 ± 4.2	8.3 ± 4.4	0.008
Measured HbA1c, %	7.8 ± 1.3	7.3 ± 1.4	0.010
TC, mmol/L	4.6 ± 1.4	4.6 ± 1.1	0.781
Triglycerides, mmol/L	1.9 ± 1.6	1.9 ± 1.4	0.502
HDL-C, mmol/L	1.1 ± 0.3	1.1 ± 0.2	0.417
LDL-C, mmol/L	2.8 ± 0.9	2.9 ± 0.8	0.412
Uric acid, umol/L	342.3 ± 112.2	321.6 ± 100.3	0.002
eGFR, mL/min	79.4 ± 36.7	86.5 ± 37.4	0.008
Coronary angiography			
GPIIb/IIIa antagonists	121 (33.8)	237 (33.0)	0.515
Anticoagulation	33 (8.8)	65 (9.1)	0.482
Lesion vessels	3.3 ± 1.5	2.9 ± 1.5	< 0.001
Three-vessel disease	213 (56.8)	348 (48.5)	< 0.001
Number of stents	1.0 ± 0.49	1.12 ± 0.50	< 0.001
Gensini score, (IQR)	73.1 (45.3–94.8)	67.0 (36.3–89.8)	0.035
Echocardiography			
LVEF	0.53 ± 0.12	0.55 ± 0.11	0.037
Medications use at discharge			
Aspirin	357 (95.2)	693 (96.7)	0.154
Clopidogrel/Ticagrelor	369 (98.4)	708 (98.7)	0.414
Statin	366 (97.6)	687 (95.8)	0.088
Beta blockers	301 (80.3)	576 (80.3)	0.477
ACEI/ARB	240 (64.0)	462 (64.4)	0.469

Data are presented as the IQR, mean ± SD or n (%)

*BMI* body mass index, *SBP* systolic blood pressure, *DBP* diastolic blood pressure, *AMI* acute myocardial infarction, *IQR* interquartile range, *hs-CRP* hypersensitive C-reactive protein, *FPG* fasting plasma glucose, *TC* total cholesterol, *HDL-C* high-density lipoprotein cholesterol, *LDL-C* low-density lipoprotein cholesterol, *SCr* serum creatinine concentration, *eGFR* estimated glomerular filtration rate, *LVEF* left ventricular ejection fraction, *ACEI* angiotensin converting enzyme inhibitor, *ARB* angiotensin receptor blocker

Univariate and multivariate analyses and predictors for MACCEs within 1 year after PCI are presented in Table 3. Univariate logistic regression showed that TyG index, age, Killip class > 1, smoking history, hypertension, DM, anaemia, three-vessel disease, DBP, white blood cells, platelets, albumin, measured HbA1c, uric acid, eGFR, lesion vessels, number of stents and LVEF were risk factors for MACCEs in STEMI patients after PCI (all P < 0.05). Co-linearity analysis of MACCEs predictors and TyG index are presented in Table 4. Co-linearity analysis showed that hypertension, diabetes, HbA1c, FPG and TyG index had high co-linearity. Therefore, hypertension, diabetes, HbA1c and FPG were not included in the multivariate model. After adjusting for age and other potential confounding factors, multivariate logistic regression showed that the TyG index, Killip class > 1, anaemia, albumin, uric acid, number of stents and LVEF were independent predictors of MACCEs in STEMI patients after PCI (all P < 0.05).

**Table 3:** Univariate and multivariate analysis and predictors of MACCEs within 1 year after PCI

Variable	Univariate analysis			Multivariate analysis		
	OR	95% CI	P value	OR	95% CI	P value
TyG index grouping						
Q1	1			1		
Q2	1.096	0.778–1.545	0.600	1.117	0.668–1.843	0.587
Q3	1.542	1.083–2.194	0.016	1.356	0.852–2.160	0.199
Q4	1.809	1.263–2.590	0.001	1.529	1.001–2.061	0.003
Age, years	1.021	1.011–1.032	< 0.001	1.011	0.992–1.031	0.233
Male	0.922	0.676–1.256	0.605			
BMI, kg/m^2^	1.005	0.950–1.043	0.460			
SBP, mmHg	1.001	0.994–1.006	0.839			
DBP, mmHg	0.990	0.981–0.999	0.029	0.993	0.982–1.005	0.258
Heart rate, bpm	0.999	0.991–1.008	0.905			
Killip class > 1	2.188	1.667–2.874	< 0.001	1.722	1.185–2.471	0.002
Smoker	1.347	1.047–1.733	0.020	1.191	0.858–1.653	0.297
Hypertension	1.631	1.250–2.127	< 0.001			
Diabetes mellitus	1.672	1.261–2.216	< 0.001			
Anemia	1.351	0.940–1.941	< 0.001	1.282	0.995–1.563	0.024
Previous AMI	1.151	0.499–2.655	0.742			
Atrial fibrillation	0.864	0.463–1.611	0.645			
Biochemical indicators						
NT-proBNP, pg/mL (IQR)	1.057	0.909–1.158	0.770			
Cardiac troponin I, ng/ml	1.018	0.830–1.257	0.571			
hs-CRP, mg/L (IQR)	1.026	0.975–1.074	0.302			
White blood cells, 10^9^/L	1.037	1.001–1.075	0.047	1.038	0.988–1.091	0.141
Neutrophil ratio	0.998	0.988–1.008	0.680			
Platelet, 10^9^/L	1.002	1.000–1.004	0.025	1.002	0.999–1.004	0.285
Albumin, g/L	0.971	0.947–0.996	0.023	0.963	0.928–1.000	0.050
Measured HbA1c, %	1.173	1.042–1.321	0.005			
FPG, mmol/L	1.040	1.010–1.072	0.009			
TC, mmol/L	1.015	0.916–1.124	0.781			
Triglycerides, mmol/L	1.021	0.909–1.131	0.503			
HDL-C, mmol/L	0.813	0.494–1.339	0.813			
LDL-C, mmol/L	0.938	0.806–1.092	0.412			
Uric acid, umol/L	1.002	1.001–1.003	0.002	1.002	1.000–1.004	0.013
eGFR, mL/min	0.995	0.991–0.999	0.009	0.999	0.993–1.005	0.751
Coronary angiography						
GPIIb/IIIa antagonists	0.910	0.703–1.176	0.470			
Anticoagulation	1.406	0.773–2.557	0.264			
Lesion vessels	1.163	1.071–1.263	< 0.001	1.092	0.960–1.243	0.180
Three-vessel disease	1.394	1.084–1.793	0.010	1.244	0.830–1.865	0.289
Number of stents	1.575	1.220–2.032	< 0.001	1.709	1.229–2.375	0.001
Gensini score	1.008	1.002–1.017	0.022	1.002	0.993–1.010	0.366
LVEF	0.303	0.099–0.931	0.037	0.121	0.029–0.057	0.004
Medications use at discharge						
Aspirin	0.687	0.368–1.282	0.238			
Clopidogrel/ticagrelor	0.782	0.276–2.213	0.643			
Statin	1.776	0.834–3.781	0.136			
Beta blockers	0.979	0.716–1.339	0.895			
ACEI/ARB	1.208	0.917–1.590	0.179			

Co-linearity analysis showed that hypertension, diabetes, HbA1c, FPG and TyG index had high co-linearity. Therefore, hypertension, diabetes, HbA1c and FPG weren’t included in multivariate model

*BMI* body mass index, *SBP* systolic blood pressure, *DBP* diastolic blood pressure, *AMI* acute myocardial infarction, *IQR* interquartile range, *hs-CRP* hypersensitive C-reactive protein, *TC* total cholesterol, *HDL-C* high-density lipoprotein cholesterol, *LDL-C* low-density lipoprotein cholesterol, *SCr* Serum creatinine concentration, *eGFR* estimated glomerular filtration rate, *LVEF* left ventricular ejection fraction, *ACEI* angiotensin converting enzyme inhibitor, *ARB* angiotensin receptor blocker

**Table 4:** Co-linearity analysis of MACCEs predictors and TyG index

	Unstandardized coefficients		Standardized coefficients	t	Sig.	Collinearity statistics	
	B	Std. error	Beta			Tolerance	VIF
(Constant)	6.683	0.356		18.758	0.000		
Age, years	− 0.006	0.002	− 0.100	− 2.406	0.016	0.424	2.361
DBP, mmHg	0.005	0.001	0.098	3.231	0.001	0.801	1.248
Killip class > 1	− 0.080	0.056	− 0.044	− 1.415	0.157	0.754	1.325
Smoker	0.094	0.043	0.066	2.184	0.029	0.803	1.245
Hypertension	0.066	0.063	0.206	1.050	0.298	0.097	10.331
Diabetes mellitus	0.287	0.371	0.207	0.772	0.444	0.052	19.393
Anemia	− 0.041	0.066	− 0.018	− 0.617	0.538	0.824	1.214
White blood cells	− 0.012	0.006	− 0.061	− 1.931	0.054	0.741	1.349
Platelet	0.001	0.003	0.111	3.700	0.000	0.861	1.225
Albumin	0.021	0.005	0.137	4.461	0.000	0.861	1.225
Measured HbA1c	− 0.053	0.024	− 0.447	− 2.167	0.035	0.087	11.464
FPG	0.545	0.098	1.083	5.576	0.000	0.098	10.171
Uric acid	0.031	0.015	0.065	2.245	0.025	0.864	1.157
eGFR	0.002	0.001	0.116	2.970	0.003	0.475	2.106
Lesion vessels	− 0.015	0.017	− 0.033	− 0.900	0.368	0.551	1.814
three-vessel disease	0.118	0.052	0.082	2.283	0.023	0.560	1.787
Number of stents	0.087	0.006	0.482	4.721	0.002	0.682	1.160
Gensini score	0.074	0.030	0.293	2.456	0.018	0.260	3.848
LVEF	0.508	0.186	0.080	2.733	0.006	0.862	1.160

Dependent variable: TyG index

**Fig. 1:**
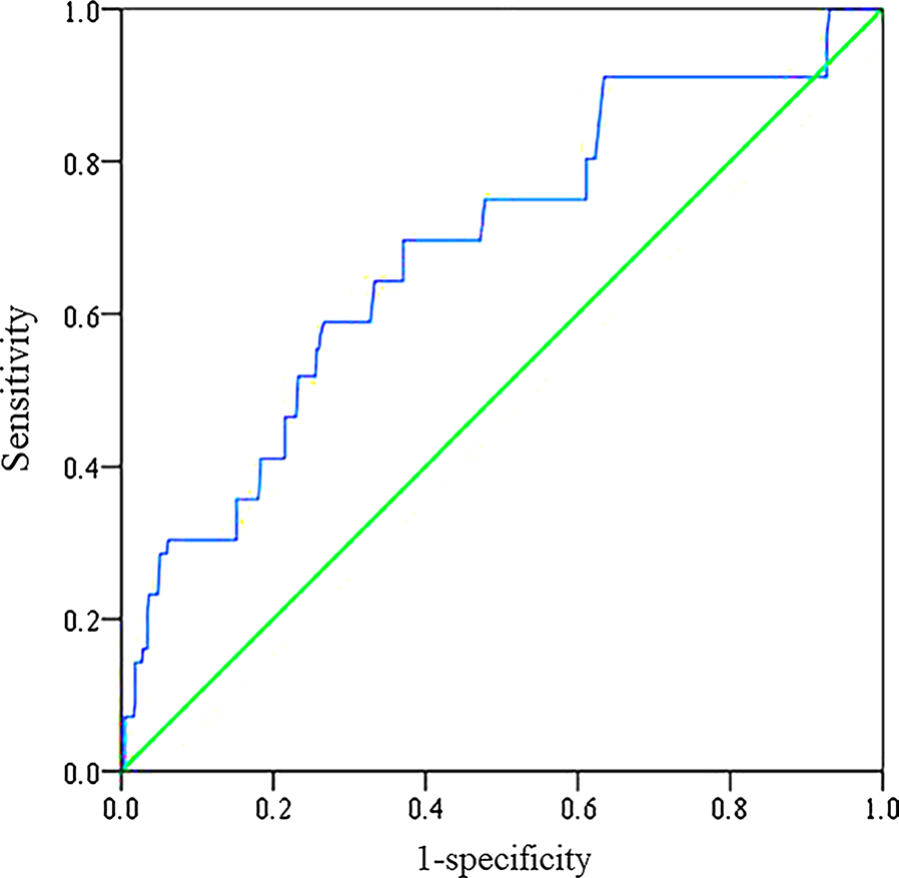
The receiver operating characteristic (ROC) curves of the triglyceride-glucose index as a marker to predict MACCEs in STEMI patients after PCI. The area under ROC curves (AUCs) of the triglyceride-glucose index for predicting the occurrence of MACCEs in STEMI patients within 1 year after PCI was 0.685 (95% CI 0.610–0.761; P = 0.001)

The ROC curves of the TyG index as a marker to predict MACCEs in STEMI patients after PCI are illustrated in Fig. 1. The AUC of the TyG index for predicting the occurrence of MACCEs in STEMI patients after PCI was 0.685 (95% CI 0.610–0.761; P = 0.001). The AUCs of the TyG index for predicting the occurrence of MACCEs after adjusting for sex and DM are shown in Table 5. The AUCs of FPG, TGs and the TyG index for predicting the occurrence of MACCEs are shown in Table 6.

**Table 5:** AUCs of the TyG index predicting the occurrence of MACCEs after adjusting for sex and DM

Variables	TyG index			
	Male	Female	DM	Non-DM
AUC (95% CI)	0.705 (0.618–0.796)	0.654 (0.588–0.679)	0.699 (0.613–0.785)	0.678 (0.611–0.746)
P value	0.008	0.019	0.001	0.015

*DM* diabetes mellitus, *TyG index* the triglyceride–glucose index

**Table 6:** AUCs of FPG, TGs and TyG index predicting the occurrence of MACCEs

Variables	AUC (95% CI)	P value
FPG	0.642 (0.546–0.738)	0.011
TG	0.549 (0.483–0.614)	0.217
TyG index	0.685 (0.610–0.761)	0.001

*FPG* fasting plasma glucose, *TG* triglycerides, *TyG index* the triglyceride–glucose index

**Fig. 2:**
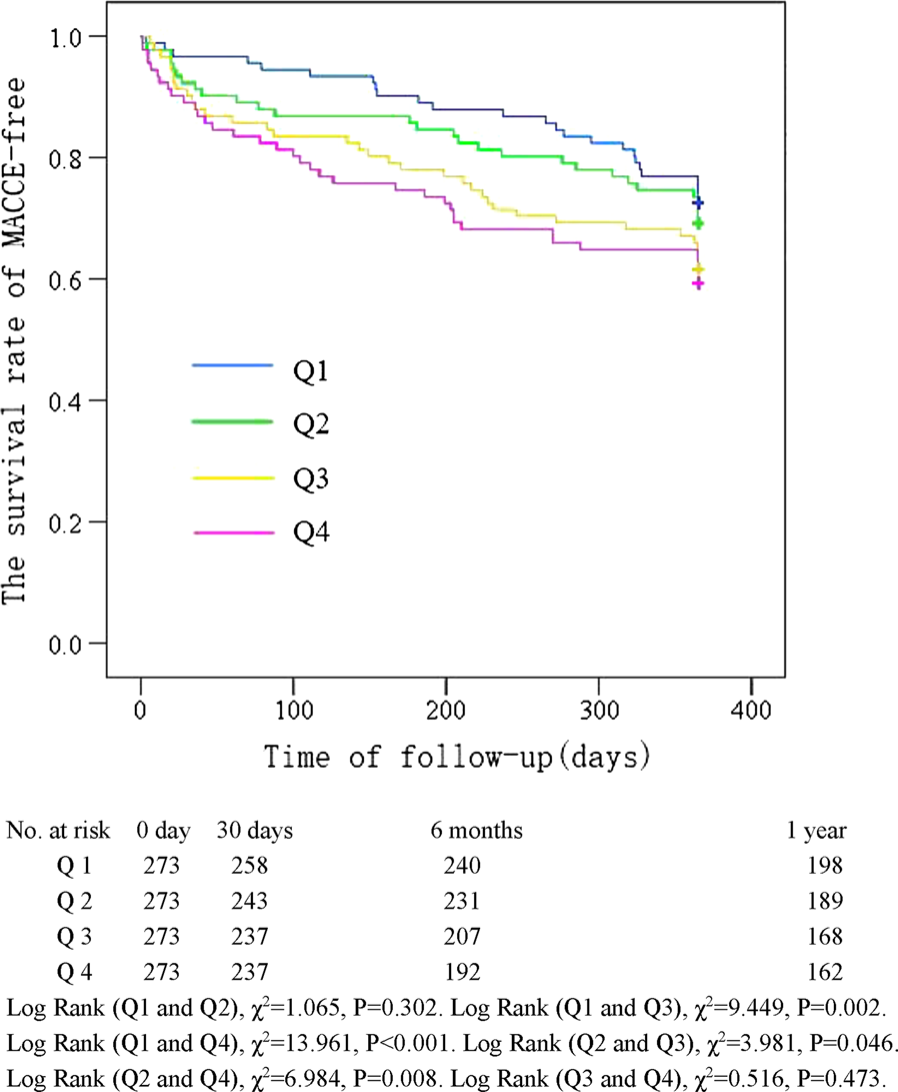
The follow-up without a MACCE (MACCE-free) survival curve of 4 groups. The incidence of MACCEs within 30 days, 6 months and 1 year after PCI was higher among STEMI patients with TyG index levels in the highest quartile (Q4)

**Fig. 3:**
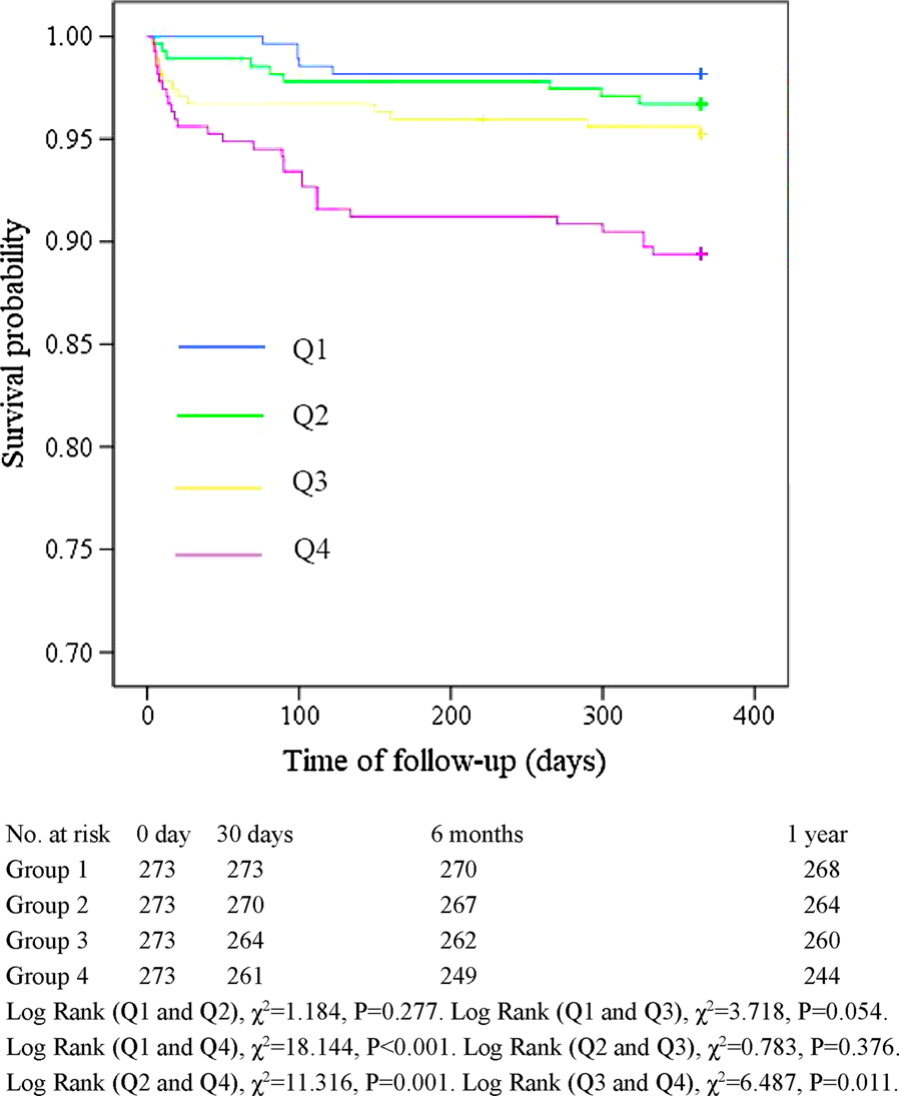
Cumulative probability of overall survival of 4 groups during the 1-year follow-up. The incidence of all-cause mortality within 30 days, 6 months and 1 year after PCI was higher among STEMI patients with TyG index levels in the highest quartile (Q4)

**Fig. 4:**
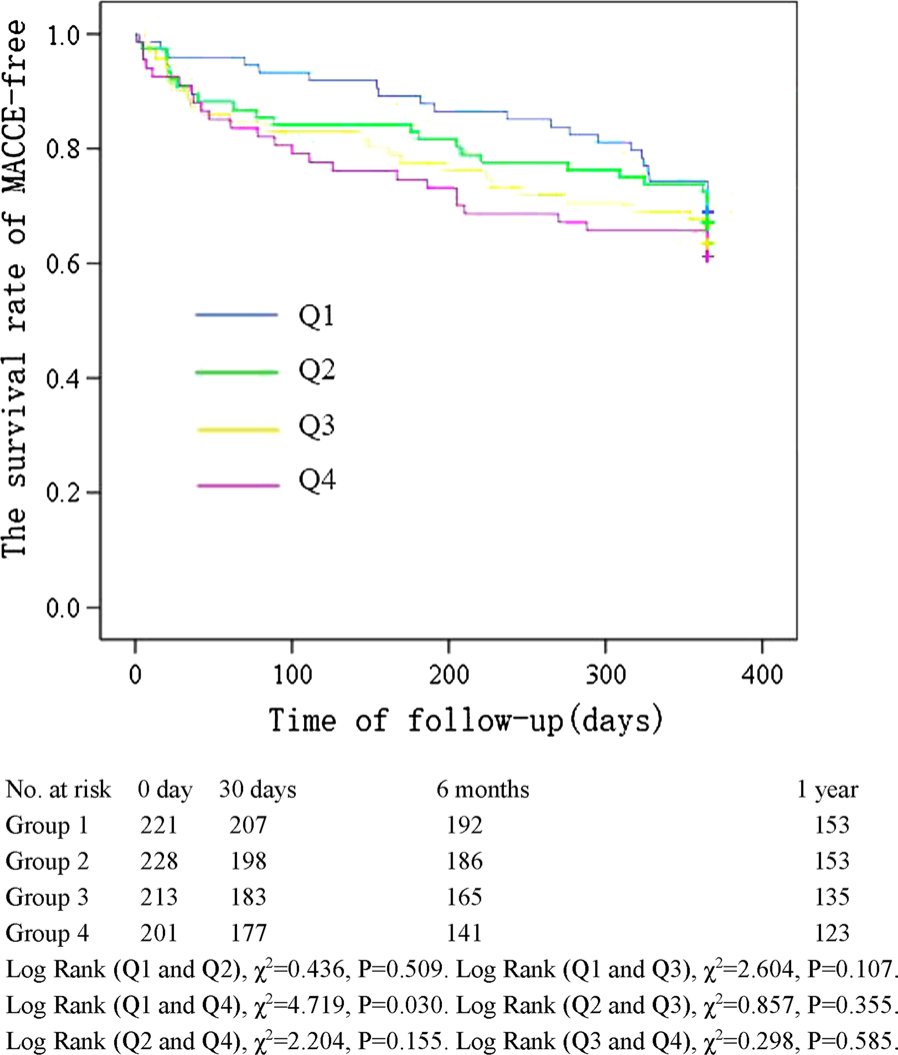
The follow-up without a MACCE (MACCE-free) survival curve of 4 groups of male patients. The incidence of MACCEs within 30 days, 6 months and 1 year after PCI was higher among male STEMI patients with TyG index levels in the highest quartile (Q4)

**Fig. 5:**
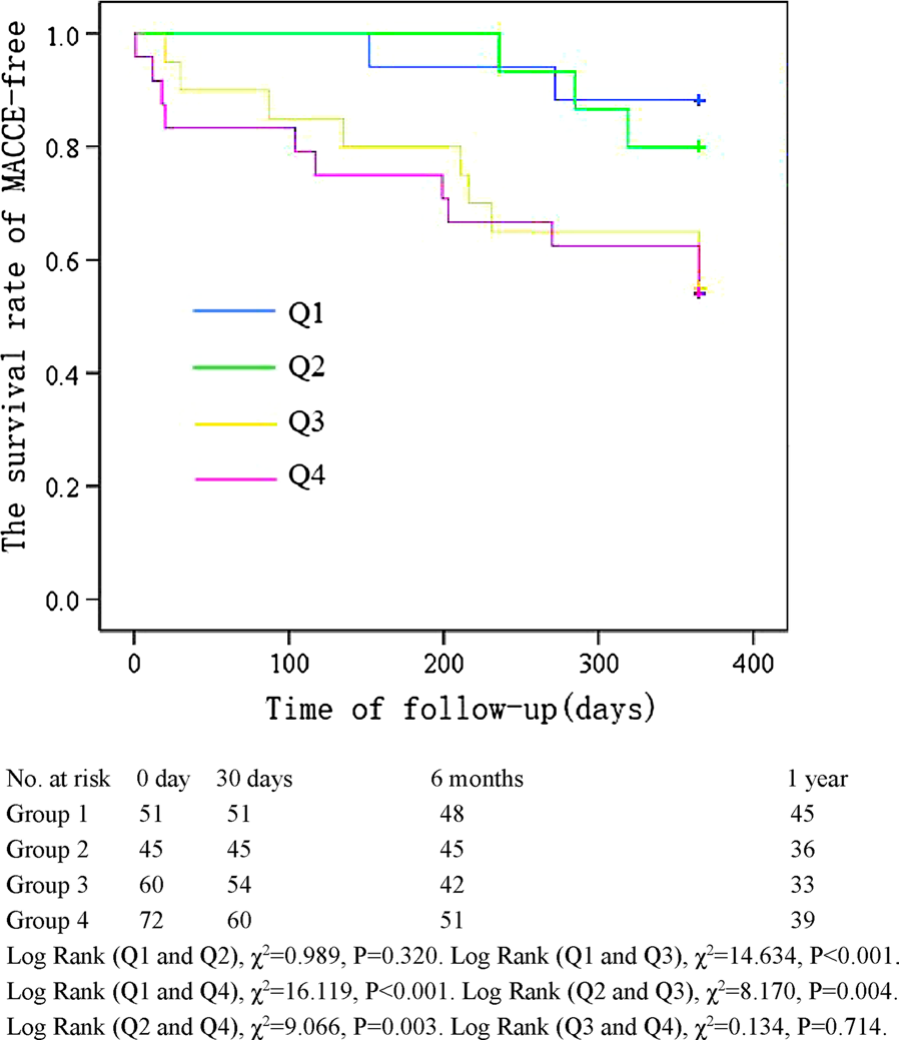
The follow-up without a MACCE (MACCE-free) survival curve of 4 groups of female patients. The incidence of MACCEs within 30 days, 6 months and 1 year after PCI was higher among female STEMI patients with TyG index levels in the highest quartile (Q4)

The Kaplan–Meier curve showing the follow-up without a MACCE (MACCE-free) survival curve of each TyG index group is illustrated in Fig. 2. The cumulative probability of overall survival of the 4 groups at the 1-year follow-up is illustrated in Fig. 3. The MACCE-free survival curve of 4 groups of male patients is illustrated in Fig. 4, and the MACCE-free survival curve of 4 groups of female patients is illustrated in Fig. 5. The incidence of MACCEs and all-cause mortality within 30 days, 6 months and 1 year after PCI were higher among STEMI patients with TyG index levels in the highest quartile (Q4).

## Discussion

In this study, we investigated the prognostic role of the TyG index in STEMI patients undergoing PCI. This study, for the first time, demonstrated that the incidence of MACCEs and all-cause mortality within 30 days, 6 months and 1 year after PCI was higher among STEMI patients with TyG index levels in the highest quartile. After adjusting for the confounding factors, the TyG index was significantly associated with an increased risk of MACCEs in STEMI patients within 1 year after PCI, with a value of 1.529 (95% CI 1.001–2.061; P = 0.003) for those in the highest quartile. In addition, the ROC curve showed that the TyG index had a high predictive value for MACCEs in STEMI patients.

IR is defined as a clinical or experimental condition in which insulin exerts a lower biological effect than expected. IR can induce an imbalance in glucose metabolism that generates chronic hyperglycaemia, which in turn triggers oxidative stress and causes an inflammatory response that leads to cell damage. Moreover, IR can also alter systemic lipid metabolism, which then leads to the development of dyslipidaemia. Overall, IR contributes to the development of CVD primarily via two independent pathways: (1) atheroma plaque formation and (2) ventricular hypertrophy and diastolic abnormality [27]. Interestingly, a strong correlation between IR and the risk of developing CVD has been established [28]. The Bruneck study revealed that IR is associated with subsequent symptomatic CVD independent of traditional risk factors in the general population [7]. Eddy et al. showed that IR is likely the most significant single cause of coronary artery disease [29].

The TyG index is a composite indicator composed of TGs and FBG, has been demonstrated to be a good marker of IR and has a high sensitivity and specificity for identifying metabolic syndrome [30]. Several studies have documented the TyG index as a surrogate for identifying IR [11, 12, 31, 32]. In addition, it has been reported that the TyG index might be useful for the early identification of apparently healthy individuals at high risk of developing cardiovascular events [18]. The findings of Won et al. showed that the TyG index is significantly associated with the presence and severity of CAD and severe coronary calcification [33]. Alessandra et al. showed that the TyG index was positively associated with a higher prevalence of symptomatic CAD and could be used as a marker of atherosclerosis [17]. Lee et al. showed that a higher TyG index is associated with an increased risk of coronary artery stenosis in asymptomatic subjects with type 2 diabetes, particularly when they have risk factors for cardiovascular disease [34]. The findings of Jin et al. showed that the TyG index was positively associated with future cardiovascular events, suggesting that the TyG index may be a useful marker for predicting clinical outcomes in patients with CAD and that the TyG index might have better prognostic value than haemoglobin glycation indexes (HGIs) in diabetes patients with new-onset, stable CAD [35, 36]. A recent study showed that the TyG index might be an independent predictor of coronary artery disease severity and cardiovascular outcomes in non-ST-segment elevation acute coronary syndrome [37]. However, no data are currently available regarding the effects of the TyG index on clinical outcomes in STEMI patients undergoing PCI. Our study indicated an association between higher TyG index levels and an increased risk of MACCEs for the first time, and the TyG index might be a valid predictor of clinical outcomes in STEMI patients undergoing PCI. Xue et al. found substantial similarities in the inflammatory profiles associated with diabetes and CVD [29]. The mechanisms underlying the close connection between the TyG index and CVD may be attributed to systemic inflammation, oxidative stress, endothelial dysfunction, and vascular remodelling mediated by IR [6, 30, 31, 38]. In addition, Zhang et al. found that the risk of incident diabetes was increased with increasing TyG index among rural Chinese people, and the index might be an important indicator to identify people at high risk of diabetes [39]. Moreover, there were also studies showing that the TyG index may help select people at early risk of future stroke and hypertension without other strong independent risk factors [19, 21]. However, more efforts need to be made to clarify the exact mechanisms of the association between the TyG index and CVD, stroke, hypertension and metabolic disorders and to provide ideas for improving risk stratification.

## Study limitations

The following limitations of the present study should be addressed. First, the findings are restricted to a selected group of Chinese patients from one centre, and the follow-up time might not be long enough. Second, the use of hypoglycaemic treatment was not recorded, and the changes in the TyG index during the follow-up period were not measured or analysed. Third, other confounding factors, such as cardiorespiratory fitness, nutritional data and exercise habits, were not included in the model. A larger sample size, longer follow-up time, and multi-centre trials are necessary to confirm our findings.

## Conclusion

In conclusion, the current study first demonstrated that higher TyG index values represent a strong independent predictor of an increased risk of MACCEs in STEMI patients within 1 year after PCI. In addition, Killip class > 1, anaemia, albumin, uric acid, number of stents and LVEF were independent predictors of MACCEs in STEMI patients within 1 year after PCI. Based on these strong results, the TyG index might be a simple, easy-to-use, reliable parameter to predict the prognosis of STEMI patients and to provide ideas for improving STEMI risk stratification.

## Data Availability

The datasets used and/or analyzed in the study are available from the corresponding author upon reasonable request.

## Abbreviations

IR: insulin resistance
TyG index: triglyceride–glucose index
STEMI: acute ST-elevation myocardial infarction
PCI: percutaneous coronary intervention
ACS: percutaneous coronary intervention
CVD: cardiovascular disease
MACCE: major adverse cardiac and cerebrovascular events
HOMA-IR: homeostasis model assessment of insulin resistance
BMI: body mass index
SBP: systolic blood pressure
DBP: diastolic blood pressure
AMI: acute myocardial infarction
IQR: interquartile range
hs-CRP: hypersensitive C-reactive protein
FPG: fasting plasma glucose
TC: total cholesterol
HDL-C: high-density lipoprotein cholesterol
LDL-C: low-density lipoprotein cholesterol
SCr: serum creatinine concentration
eGFR: estimated glomerular filtration rate
LVEF: left ventricular ejection fraction

## References

[1] Roger VL, Go AS, Lloyd-Jones DM (2012). Heart disease and stroke statistics—2012 update: a report from the American Heart Association. Circulation.

[2] Fox KA, Carruthers KF, Dunbar DR (2010). Underestimated and under-recognized: the late consequences of acute coronary syndrome (GRACE UK-Belgian Study). Eur Heart J.

[3] Fox KA, Cokkinos DV, Deckers J, Keil U, Maggioni A, Steg G (2000). The ENACT study: a pan-European survey of acute coronary syndromes. European Network for Acute Coronary Treatment. Eur Heart J.

[4] Fox KA, Goodman SG, Anderson FJ, Granger CB, Moscucci M, Flather MD, Spencer F, Budaj A, Dabbous OH, Gore JM (2003). From guidelines to clinical practice: the impact of hospital and geographical characteristics on temporal trends in the management of acute coronary syndromes. The Global Registry of Acute Coronary Events (GRACE). Eur Heart J.

[5] Laakso M (2015). Is insulin resistance a feature of or a primary risk factor for cardiovascular disease?. Curr Diab Rep.

[6] Laakso M, Kuusisto J (2014). Insulin resistance and hyperglycaemia in cardiovascular disease development. Nat Rev Endocrinol.

[7] Bonora E, Kiechl S, Willeit J, Oberhollenzer F, Egger G, Meigs JB, Bonadonna RC, Muggeo M (2007). Insulin resistance as estimated by homeostasis model assessment predicts incident symptomatic cardiovascular disease in caucasian subjects from the general population: the Bruneck study. Diabetes Care.

[8] Yusuf S, Hawken S, Ounpuu S, Bautista L, Franzosi MG, Commerford P, Lang CC, Rumboldt Z, Onen CL, Lisheng L, Tanomsup S, Wangai PJ, Razak F, Sharma AM, Anand SS (2005). Obesity and the risk of myocardial infarction in 27,000 participants from 52 countries: a case–control study. Lancet.

[9] Du T, Yuan G, Zhang M, Zhou X, Sun X, Yu X (2014). Clinical usefulness of lipid ratios, visceral adiposity indicators, and the triglycerides and glucose index as risk markers of insulin resistance. Cardiovasc Diabetol.

[10] Navarro-Gonzalez D, Sanchez-Inigo L, Pastrana-Delgado J, Fernandez-Montero A, Martinez JA (2016). Triglyceride-glucose index (TyG index) in comparison with fasting plasma glucose improved diabetes prediction in patients with normal fasting glucose: the vascular-metabolic CUN cohort. Prev Med.

[11] Guerrero-Romero F, Simental-Mendia LE, Gonzalez-Ortiz M, Martinez-Abundis E, Ramos-Zavala MG, Hernandez-Gonzalez SO, Jacques-Camarena O, Rodriguez-Moran M (2010). The product of triglycerides and glucose, a simple measure of insulin sensitivity. Comparison with the euglycemic-hyperinsulinemic clamp. J Clin Endocrinol Metab.

[12] Simental-Mendia LE, Rodriguez-Moran M, Guerrero-Romero F (2008). The product of fasting glucose and triglycerides as surrogate for identifying insulin resistance in apparently healthy subjects. Metab Syndr Relat Disord.

[13] Lee SB, Ahn CW, Lee BK, Kang S, Nam JS, You JH, Kim MJ, Kim MK, Park JS (2018). Association between triglyceride glucose index and arterial stiffness in Korean adults. Cardiovasc Diabetol.

[14] Zhang M, Wang B, Liu Y (2017). Cumulative increased risk of incident type 2 diabetes mellitus with increasing triglyceride glucose index in normal-weight people: the Rural Chinese Cohort Study. Cardiovasc Diabetol.

[15] Kim MK, Ahn CW, Kang S, Nam JS, Kim KR, Park JS (2017). Relationship between the triglyceride glucose index and coronary artery calcification in Korean adults. Cardiovasc Diabetol.

[16] Irace C, Carallo C, Scavelli FB, De Franceschi MS, Esposito T, Tripolino C, Gnasso A (2013). Markers of insulin resistance and carotid atherosclerosis. A comparison of the homeostasis model assessment and triglyceride glucose index. Int J Clin Pract.

[17] Da SA, Caldas A, Hermsdorff H (2019). Triglyceride-glucose index is associated with symptomatic coronary artery disease in patients in secondary care. Cardiovasc Diabetol.

[18] Sanchez-Inigo L, Navarro-Gonzalez D, Fernandez-Montero A, Pastrana-Delgado J, Martinez JA (2016). The TyG index may predict the development of cardiovascular events. Eur J Clin Invest.

[19] Sanchez-Inigo L, Navarro-Gonzalez D, Fernandez-Montero A (2017). Risk of incident ischemic stroke according to the metabolic health and obesity states in the vascular-metabolic CUN cohort. Int J Stroke.

[20] Sanchez-Inigo L, Navarro-Gonzalez D, Fernandez-Montero A (2016). The TyG index may predict the development of cardiovascular events. Eur J Clin Invest.

[21] Sanchez-Inigo L, Navarro-Gonzalez D, Pastrana-Delgado J (2016). Association of triglycerides and new lipid markers with the incidence of hypertension in a Spanish cohort. J Hypertens.

[22] Chinese Society of Cardiology (2010). Guidelines for the diagnosis and treatment of acute ST segment elevation myocardial infarction in 2010 (China). Chin J Cardiol.

[23] Guerrero-Romero F, Simental-Mendia LE, Gonzalez-Ortiz M (2010). The product of triglycerides and glucose, a simple measure of insulin sensitivity. Comparison with the euglycemic-hyperinsulinemic clamp. J Clin Endocrinol Metab.

[24] Liu LS (2011). 2010 Chinese guidelines for the management of hypertension. Zhonghua Xin Xue Guan Bing Za Zhi.

[25] Chinese Diabetes Society (2014). China’s prevention and treatment guideline for type 2 diabetes Mellitus (2013 edition). Chin J Diab Mellitus.

[26] Killip TR, Kimball JT (1967). Treatment of myocardial infarction in a coronary care unit. A 2 year experience with 250 patients. Am J Cardiol.

[27] Ormazabal V, Nair S, Elfeky O, Aguayo C, Salomon C, Zuniga FA (2018). Association between insulin resistance and the development of cardiovascular disease. Cardiovasc Diabetol.

[28] Gast KB, Tjeerdema N, Stijnen T, Smit JW, Dekkers OM (2012). Insulin resistance and risk of incident cardiovascular events in adults without diabetes: meta-analysis. PLoS ONE.

[29] Eddy D, Schlessinger L, Kahn R, Peskin B, Schiebinger R (2009). Relationship of insulin resistance and related metabolic variables to coronary artery disease: a mathematical analysis. Diabetes Care.

[30] Angoorani P, Heshmat R, Ejtahed HS, Motlagh ME, Ziaodini H, Taheri M, Aminaee T, Goodarzi A, Qorbani M, Kelishadi R (2018). Validity of triglyceride-glucose index as an indicator for metabolic syndrome in children and adolescents: the CASPIAN-V study. Eat Weight Disord.

[31] Bastard JP, Lavoie ME, Messier V, Prud’Homme D, Rabasa-Lhoret R (2012). Evaluation of two new surrogate indices including parameters not using insulin to assess insulin sensitivity/resistance in non-diabetic postmenopausal women: a MONET group study. Diabetes Metab.

[32] Vasques AC, Novaes FS, de Oliveira MS, Souza JR, Yamanaka A, Pareja JC, Tambascia MA, Saad MJ, Geloneze B (2011). TyG index performs better than HOMA in a Brazilian population: a hyperglycemic clamp validated study. Diabetes Res Clin Pract.

[33] Won KB, Kim YS, Lee BK, Heo R, Han D, Lee JH, Lee SE, Sung JM, Cho I, Park HB, Cho IJ, Chang HJ (2018). The relationship of insulin resistance estimated by triglyceride glucose index and coronary plaque characteristics. Medicine (Baltimore).

[34] Lee EY, Yang HK, Lee J, Kang B, Yang Y, Lee SH, Ko SH, Ahn YB, Cha BY, Yoon KH (2016). Triglyceride glucose index, a marker of insulin resistance, is associated with coronary artery stenosis in asymptomatic subjects with type 2 diabetes. Lipids Health Dis.

[35] Jin JL, Cao YX, Wu LG, You XD, Guo YL, Wu NQ, Zhu CG, Gao Y, Dong QT, Zhang HW (2018). Triglyceride glucose index for predicting cardiovascular outcomes in patients with coronary artery disease. J Thorac Dis.

[36] Jin JL, Sun D, Cao YX, Guo YL, Wu NQ, Zhu CG, Gao Y, Dong QT, Zhang HW, Liu G (2018). Triglyceride glucose and haemoglobin glycation index for predicting outcomes in diabetes patients with new-onset, stable coronary artery disease: a nested case-control study. Ann Med.

[37] Mao Q, Zhou D, Li Y, Wang Y, Xu SC, Zhao XH (2019). The triglyceride-glucose index predicts coronary artery disease severity and cardiovascular outcomes in patients with non-st-segment elevation acute coronary syndrome. Dis Markers.

[38] Yang SW, Park KH, Zhou YJ (2016). The impact of hypoglycemia on the cardiovascular system: physiology and pathophysiology. Angiology.

[39] Bao X, Borne Y, Johnson L, Muhammad IF, Persson M, Niu K, Engstrom G (2018). Comparing the inflammatory profiles for incidence of diabetes mellitus and cardiovascular diseases: a prospective study exploring the ‘common soil’ hypothesis. Cardiovasc Diabetol.

